# Identification of Key Phospholipids That Bind and Activate Atypical PKCs

**DOI:** 10.3390/biomedicines9010045

**Published:** 2021-01-06

**Authors:** Suresh Velnati, Sara Centonze, Federico Girivetto, Daniela Capello, Ricardo M. Biondi, Alessandra Bertoni, Roberto Cantello, Beatrice Ragnoli, Mario Malerba, Andrea Graziani, Gianluca Baldanzi

**Affiliations:** 1 Department of Translational Medicine, University of Piemonte Orientale, 28100 Novara, Italy; sara.centonze@uniupo.it (S.C.); 20020549@studenti.uniupo.it (F.G.); daniela.capello@uniupo.it (D.C.); alessandra.bertoni@med.uniupo.it (A.B.); roberto.cantello@med.uniupo.it (R.C.); mario.malerba@uniupo.it (M.M.); gianluca.baldanzi@med.uniupo.it (G.B.); 2 Center for Translational Research on Allergic and Autoimmune Diseases (CAAD), University of Piemonte Orientale, 28100 Novara, Italy; 3 UPO Biobank, University of Piemonte Orientale, 28100 Novara, Italy; 4 Department of Internal Medicine 1, Goethe University Hospital Frankfurt, 60590 Frankfurt, Germany; dabiondi@yahoo.co.uk; 5 Biomedicine Research Institute of Buenos Aires—CONICET—Partner Institute of the Max Planck Society, Buenos Aires C1425FQD, Argentina; 6 Respiratory Unit, Sant’Andrea Hospital, 13100 Vercelli, Italy; beatrice.ragnoli@hotmail.it; 7 Molecular Biotechnology Center, Department of Molecular Biotechnology and Health Sciences, University of Torino, 10126 Turin, Italy; andrea.graziani@unito.it; 8 Division of Oncology, Università Vita-Salute San Raffaele, 20132 Milan, Italy

**Keywords:** membrane, lipid-protein interaction, lipid signalling, kinase regulation, phosphatidylinositols

## Abstract

PKCζ and PKCι/λ form the atypical protein kinase C subgroup, characterised by a lack of regulation by calcium and the neutral lipid diacylglycerol. To better understand the regulation of these kinases, we systematically explored their interactions with various purified phospholipids using the lipid overlay assays, followed by kinase activity assays to evaluate the lipid effects on their enzymatic activity. We observed that both PKCζ and PKCι interact with phosphatidic acid and phosphatidylserine. Conversely, PKCι is unique in binding also to phosphatidylinositol-monophosphates (e.g., phosphatidylinositol 3-phosphate, 4-phosphate, and 5-phosphate). Moreover, we observed that phosphatidylinositol 4-phosphate specifically activates PKCι, while both isoforms are responsive to phosphatidic acid and phosphatidylserine. Overall, our results suggest that atypical Protein kinase C (PKC) localisation and activity are regulated by membrane lipids distinct from those involved in conventional PKCs and unveil a specific regulation of PKCι by phosphatidylinositol-monophosphates.

## 1. Introduction

Protein kinase C (PKC) is a family of multidomain Ser/Thr kinases that regulate cell growth, differentiation, apoptosis, and motility. Considering their protein structure and their biochemical characteristics, these kinases are classified into the classical or conventional PKCs (α, β, and γ isoforms; cPKCs); the novel PKCs (δ, θ, ε, and η isoforms; nPKCs); and the atypical PKCs (ζ and λ (mouse)/ι (human) isoforms; aPKCs). In physiological conditions, both atypical PKCs play a vital role in cell polarity and signalling. Indeed, these kinases regulate the subcellular localisation of a wide range of polarity proteins by phosphorylating them [1,2]. The PAR6-PAR3-aPKCs trimeric complex is fundamental to modulate the polarity of the epithelial cells and to determinate the cell fate through the orientation of the apical/basal cell asymmetric division [3,4,5]. Both the aPKCs are also known to enhance the cell migration, invasion, and epithelial–mesenchymal transition in multiple cancer cell types [6,7,8]. However, it is fascinating to observe that the two aPKCs isotypes may have specific functions in different cancer cell types. For instance, the PKCι/λ isoform promotes cancer growth and metastasis in triple-negative breast cancers [9], while PKCζ is the isoform required for the head and neck squamous cell carcinoma growth and development [10]. Nevertheless, establishing a specific isoform contribution in tumour development is made difficult by the high degree of homology between the PKCζ and PKCι sequences and the lack of specific tools to evaluate a distinct isotype activation.

All these PKC enzymes are characterised by the presence of a kinase domain in the C-terminal region and a regulatory domain placed in the N-terminal region. cPKC regulatory domains also contain a C2 domain that binds anionic phospholipids in a calcium-dependent manner. Conversely, the nPKCs C2 domain is Ca2+-independent but still diacylglycerol (DAG)-sensitive. aPKCs do not possess a C2 domain, whereas they contain a single DAG-insensitive C1 domain. Interestingly, the aPKCs C1 domain (C1 A in cPKCs and nPKCs) is preceded by a basic pseudosubstrate region (PSR) [11]. The PSR binds at the substrate-binding site in an inactive conformation and participates in keeping the kinase inactive in the absence of second messengers. In aPKCs, the C1 domain also participates in the inhibition of the catalytic domain by interactions with the small lobe [12]. In the process of activation, the PSR and the C1 domain must be released from their interactions with the catalytic domain. aPKCs further contain a Phox and Bem1 (PB1) domain located in the N-terminus. This domain extends about 85 amino acids and binds to other PB1 domain-containing proteins, such as zeta-PKC-interacting protein (ZIP/p62), Partitioning-defective Protein 6 (PAR-6) or Mitogen-Activated Protein Kinase 5 (MAPK5) through a homologous PB1–PB1 domain interaction [13].

Although not DAG-sensitive, aPKCs are recruited to membranes upon cell stimulation through protein–protein and protein–lipid interactions [14,15,16,17,18]. The contribution of lipid binding to aPKC localisation is still obscure. Limatola et al. reported that phosphatidic acid (PA), but not other anionic phospholipids, directly binds and activates PKCζ using a gel-shift assay [19]. Building upon this, Pu et al. noted that, compared with the DAG/phorbol ester-sensitive C1 domains, the rim of the binding cleft of the aPKCs C1 domains possesses four additional positively charged arginine residues (at positions 7, 10, 11, and 20) that may be responsible for PA binding. Indeed, mutations of those residues to the corresponding residues in the PKCδ C1b domain conferred a response to phorbol ester [20]. The importance of PA for aPKC regulation is underscored by our previous findings that PA production by diacylglycerol kinase alpha (DGKα) at cell-ruffling sites recruits aPKCs at the plasma membrane where their activity is necessary for protrusion extension and cell migration [14,15,21]. PKCζ is also reported to interact with ceramide (CE), which specifically binds to and regulates its kinase activity in a biphasic manner with high- and low-affinity binding sites characterised by Bmax values of 60 and 600 nM and Kd values of 7.5 and 320 nM, respectively [22]. Using CE overlay assays with proteolytic fragments of PKCζ and vesicle-binding assays with ectopically expressed protein, Wang et al. 2009 showed that a protein fragment comprising the carboxyl-terminal 20-kDa sequence of PKCζ (amino acids 405–592, distinct from the C1 domain) bound to C16:0 ceramide [23]. This interaction with CE activates PKCζ and promotes the local proapoptotic complex formation with PAR-4 [18]. Moreover, an analogous interaction was observed also between sphingosine-1-phosphate (S1P) and PKCζ. Indeed, S1P is suggested to bind the kinase domain of PKCζ constituted by R_375_ and K_399_ and reliving an autoinhibitory constrain [24]. While the PSR and C1 domain participates in the autoinhibition of catalytic activity, the PSR was found to be key for the activation by a lipid mix [11]. NMR studies suggested that phosphatidylinositol-3,4,5-trisphosphate (PI(3,4,5)P_3_) binds directly to the basic residues in the pseudosubstrate sequence of PKCζ, displacing it from the substrate-binding site during kinase activation [25]. This finding is controversial, as later studies indicated that PI(3,4,5)P_3_ does not directly regulate PKCζ activity [26]. More recently, an interesting work by Dong and colleagues suggested a membrane-targeting mechanism based on electrostatic binding between the PI(4)P, PI(4,5)P_2_, and aPKCs PSR polybasic domains. In PKCζ, this binding requires the formation of a complex with PAR-6 through the PB1 domain and regulates localisation but not activity [27].

Although phosphorylation by phosphoinositide-dependent kinase-1 (PDK1) [28] and by mTORC2 (target of rapamycin complex 2) [29] are required for aPKC activity, it is widely considered that, upon phosphorylation (maturation), the aPKCs remain in an inactive conformation stabilised by the PSR and the C1 domain. Signalling lipids activate in vitro its kinase activity, reminiscent of conventional PKC. Activators include acidic phospholipids such PA and phosphatidylserine (PS) [19], PI(3,4,5)P_3_ [30], S1P [24], and CE [22,31]. Altogether, these shreds of evidence support the possibility of the direct regulation of PKCζ by lipids, while no information is available for PKCι, which is generally assumed to share regulatory models based on high homology with PKCζ. However, a full understanding of how lipid signalling contributes to the control of aPKC localisation and activity is made difficult by the presence of heterogeneous results obtained with different assays. In here, we used a lipid overlay assay and ELISA technique by using Cova phosphatidylinositol monophosphate (PIP) screening plates to evaluate systematically the lipid-binding specificity and luminescent kinase activity assays to assess the activation of purified human PKCζ and PKCι in the presence of various lipids. We observed that both aPKC isoforms bind to PS and PA. Conversely, only PKCι specifically associates with phosphatidylinositol monophosphates. Likewise, we found that PA and PS activate both aPKCs, while only PKCι is PI(4)P-sensitive.

## 2. Results

To explore systematically the lipid-binding properties of aPKCs, initially, we decided to perform lipid overlay assays that probe highly purified lipids spotted on solid supports to screen for lipid binding in a stable way to recombinant tagged aPKC. The lipid overlay assay technique allows to assay in parallel several lipid species and has been used extensively to study the specificity of lipid-binding domains [32]. 

### 2.1. PKCζ Selectively Binds to PA and PS

**Figure 1:**
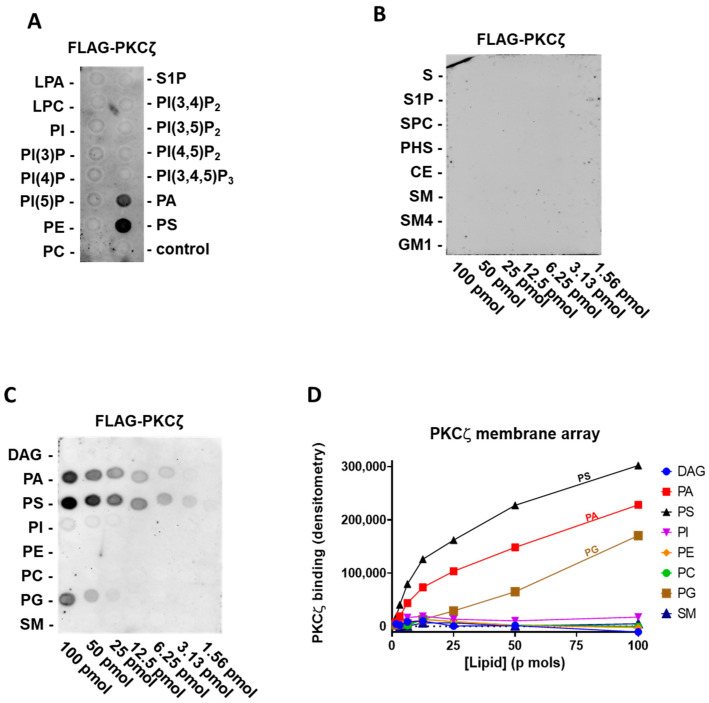
PKCζ selectively binds phosphatidic acid (PA) and phosphatidylserine (PS) and weakly to phosphatidylglycerol (PG) (**A**) Batch purified FLAG-PKCζ was incubated with a phosphatidylinositol monophosphate (PIP) strip overnight, and after washing detected with anti-FLAG antibody, a representative experiment out of three performed is shown. (**B**) Batch purified FLAG-PKCζ was incubated with a sphingo array overnight and, after washing, detected with anti-FLAG antibody. (**C**) Batch purified with FLAG-PKCζ was incubated with a membrane lipid array overnight and, after washing, detected with anti-FLAG antibody (left). (**D**) Quantification by densitometry of (**C**). DAG: diacylglycerol, PE: phosphatidylethanolamine, PC: phosphatidylcholine, SM: sphingomyelin, PKC: protein kinase C, LPA: lysophosphatidic acid, LPC: lysophosphocoline, S: sphingosine, S1P: sphingosine-1-phosphate, SPC: sphingosylphosphorycholine, PHS: phytosphingosine, CE: ceramide, SM4: sulfatide, and GM1: monosialoganglioside.

At first, we tested human FLAG-PKCζ purified by immunoprecipitation, against the main signalling lipids present in the cell membrane. Specifically, on a lipid PIP strip P-6001, that has been spotted with 100 pmol of all eight phosphoinositides (PI, PI(3)P, PI(4)P, PI (5)P, PI(3,4)P_2_, PI(3,5)P_2_, PI(4,5)P_2_, and PI(3,4,5)P_3_) and seven other biological important lipids (lysophosphatidic acid (LPA), lysophosphocoline (LPC), phosphatidylethanolamine (PE), phosphatidylcholine (PC), S1P, PA, and PS). Interestingly, we can confirm the reported interaction of PKCζ with PA and PS [19], but no direct binding to PI(3,4,5)P_3_ or other phosphatidylinositols was detected (Figure. 1A). 

Following, to establish the relative affinity for PA and PS, we used the membrane lipid array P-6003 that has been spotted with DAG, PA, PS, PI, PE, PC, phosphatidylglycerol (PG), and sphingomyelin (SM) in a concentration gradient (100–1.56 pmol). We provided evidence of selective and comparable binding to PS and PA and a very modest binding to PG (Figure 1C,D). As ceramide and S1P were reported to interact with the PKCζ C-terminal region [18], using FLAG-PKCζ, we also probed a sphingolipid array S-6001 that has been spotted with a concentration gradient of eight different sphingolipids, but we observed no specific association to any of them (Figure 1B). 

To summarise, among all the lipids tested in our assay, full-length PKCζ binds selectively to PA and PS (Figure 1 and Table 1). Conversely, we did not observe binding to any phosphatidylinositol or sphingolipids.

### 2.2. PKCι Binds to Phosphatidylinositol Monophosphates, along with PA and PS

**Figure 2:**
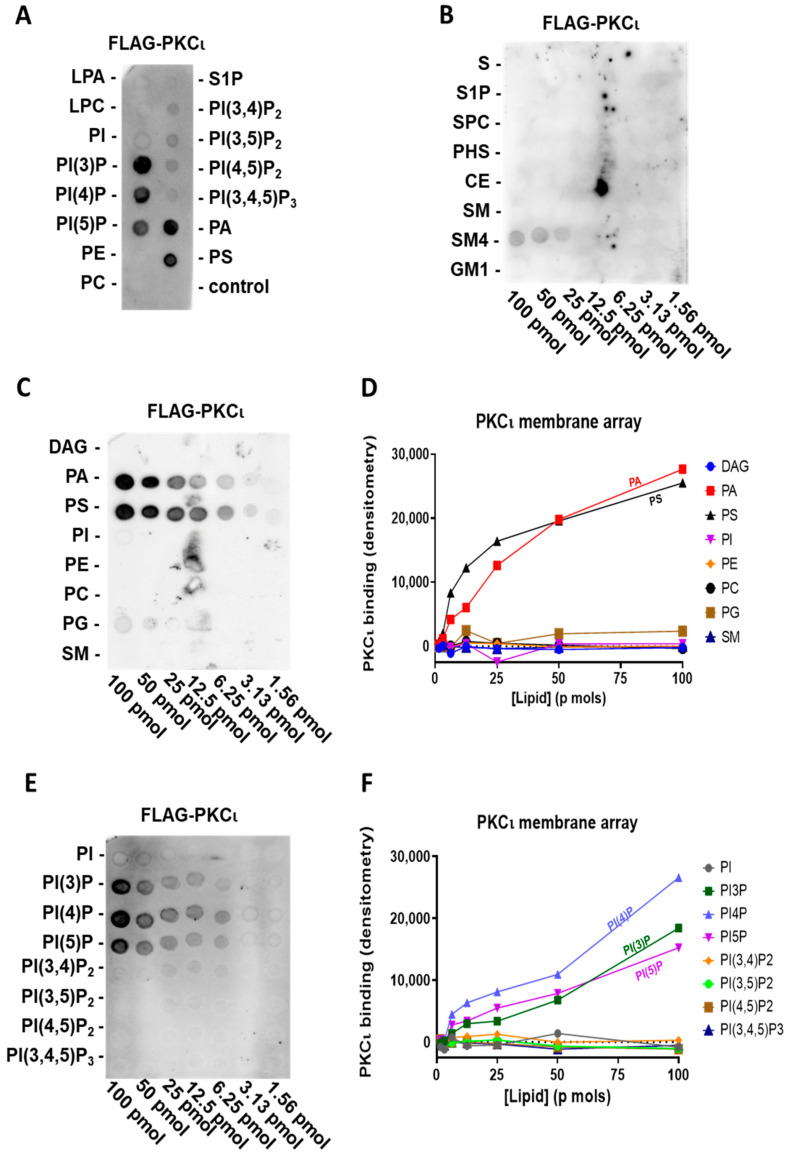
PKCι binds PIPs, PA, and PS (**A**) Highly purified FLAG-PKCι was incubated with a PIP strip overnight and, after washing, detected with anti-FLAG antibody. (**B**) Highly purified FLAG-PKCι was incubated with sphingolipid array overnight and, after washing, detected with anti-FLAG antibody. (**C**) Highly purified FLAG-PKCι was incubated with a membrane lipid array overnight and, after washing, detected with anti-FLAG antibody (left). (**D**) Quantification by densitometry of (**C**). (**E**) Highly purified FLAG-PKCι was incubated with a PIP array overnight and after, washing detected, with anti-FLAG antibody (left). (**F**) Quantification by densitometry of (**E**).

To explore the lipid-binding specificity of the highly homologous human PKCι, we used a highly purified commercial preparation of FLAG-PKCι in the same assay. Like PKCζ, we tested purified FLAG-PKCι on the previously described PIP strip P-6001, representing the main signalling lipids present in the cell membrane. We observed that PKCι also interacts with PA and PS but not with PI (Figure 2A). Surprisingly, PKCι also selectively binds to phosphatidylinositol monophosphates (PIPs, e.g., PI(3)P, PI(4)P, and PI(5)P), regardless of the phosphorylation position. Interestingly, PKCι neither binds to the phosphatidylinositol diphosphates nor triphosphates, which was already indicative of a very selective interaction mechanism.

Using the membrane lipid array P-6003 that has been spotted with DAG, PA, PS, PI, PE, PC, PG, and SM in a concentration gradient, we can confirm that also PKCι binds to PS and PA with a comparable affinity (Figure 2C,D). Moreover, to further investigate the relative affinity in PIP binding, we used a PIP array P-6100 that has been spotted with a concentration gradient of all eight phosphoinositides, i.e., PI, PI(3)P, PI(4)P, PI(5)P, PI(3,4)P_2_, PI(3,5)P_2_, PI(4,5)P_2_, and PI(3,4,5)P_3_. We confirmed the binding to phosphatidylinositol monophosphates and a lack of selectivity for the phosphate position in the PIPs (Figure 2E,F) since purified FLAG-PKCι binds to PI(3)P, PI(4)P, and PI(5)P to a similar extent. Similar to FLAG-PKCζ, we did not observe any interaction between purified FLAG-PKCι and sphingolipids, apart from a weak binding to sulfatide (SM4), which, however, could be due to its structural similarity in charge and dimensions to PIPs (Figure 2B). 

To conclude, PKCι also binds to PA and PS. Furthermore, unlike PKCζ, PKCι selectively binds to PIPs without any specificity for the phosphate position (Figure 2 and Table 1).

### 2.3. PKCι Binds to PI(3)P and PI(4)P through the Catalytic Domain

**Figure 3:**
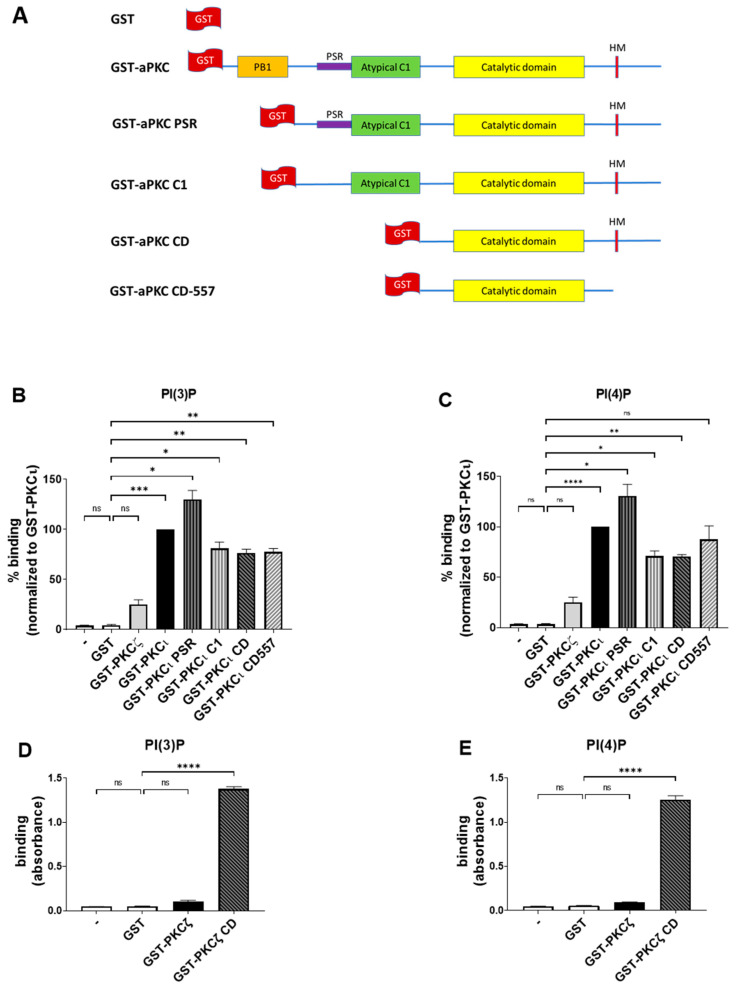
PKCι, but not PKCζ, binds selectively to both PI(3)P and PI(4)P. (**A**) Schematic domain structure of GST tagged PKCζ, PKCι, and the deletion mutants used in this study. (**B**) GST-PKCζ, GST-PKCι, and deletion mutants binding on Cova PIP screening plates coated with PI(3)P. Purified GST was used as a negative control. Data are the mean ± SEM of three independent experiments. (**C**) GST-PKCζ, GST-PKCι, and deletion mutants binding on Cova PIP screening plates coated with PI(4)P. Purified GST was used as a negative control. Data are the mean ± SEM of three independent experiments. (**D**) GST-PKCζ and GST-PKCζ CD binding on Cova PIP screening plates coated with PI(3)P. Purified GST was used as a negative control. Data are the mean ± SEM of three independent experiments. (**E**) GST-PKCζ and GST-PKCζ CD binding on Cova PIP screening plates coated with PI(4)P. Purified GST was used as a negative control. Data are the mean ± SEM of three independent experiments. A single, double, triple and four asterisks denote their significance of *p*-value ≤ 0.05, ≤ 0.01, ≤ 0.001 and ≤ 0.0001 respectively, ns mean No significant.

To further confirm the data obtained through lipid overlay assays and to identify the aPKC domains responsible for binding, we performed the ELISA technique using Cova PIP screening plates that were precoated with 20 nmols of either PI(3)P or PI(4)P per well, preblocked and ready for the addition of the proteins. On those plates, we used in-house purified GST-tagged full-length aPKCs and their deletion mutants and detected their binding using anti-GST antibodies. The constructs used are described in Figure 3A [12]. We used the same concentrations of purified GST as in the negative control.

While the GST alone gave no detectable binding, as expected, the PKCι isoform strongly bound to both PI(3)P and PI(4)P (Figure 3B,C), in line with our previous findings. Conversely, PKCζ neither bound to PI(3)P nor PI(4)P (Figure 3B,C). Those data indicate that, similar to what we observed in the lipid overlay assays, the binding of PKCι to PIPs is isoform-specific and does not require the presence of additional proteins. 

Besides, all the truncated forms of PKCι resulted in some binding to both PI(3)P and PI(4)P, suggesting that a relevant lipid binding takes place in the catalytic domain (CD), as it is the only domain common to all those truncated proteins. On the other hand, we observed an increased binding signal towards PI(3)P and PI(4)P when testing the PKCι PSR. Indeed, the PSR is a polybasic domain, enriched with Arg and Lys residues, which confers to the protein the ability to bind directly the phosphoinositides such as PI(4)P but is masked when the protein is not involved in interactions with PAR-6 [27]. It may be possible that removing the PB1 region makes the PSR domain more accessible to the electrostatic binding to the lipids, resulting in a stronger signal when compared to the full-length protein.

Moreover, we observed a strong binding of both PI(3)P and PI(4)P to PKCζ CD, a truncated mutant lacking the N-terminal PB1, PSR, and C1 domains. Interestingly, the full-length PKCζ remained unbound (Figure 3D,E), indicating that the N-terminal domains inhibited the interaction and that the PI(3)P and PI(4)P interacting region is located within evolutionarily conserved regions in the CD region of PKCζ and PKCι.

Overall, these data indicate that full-length PKCι readily binds selectively to PI(3)P and PI(4)P, while the CD appears as a primary binding site for phosphatidylinositol monophosphates. In PKCζ, this binding is masked by the presence of N-terminal regulatory domains.

### 2.4. PS and PA Activates Both aPKCs, While PI(4)P Activates PKCι Selectively

**Figure 4:**
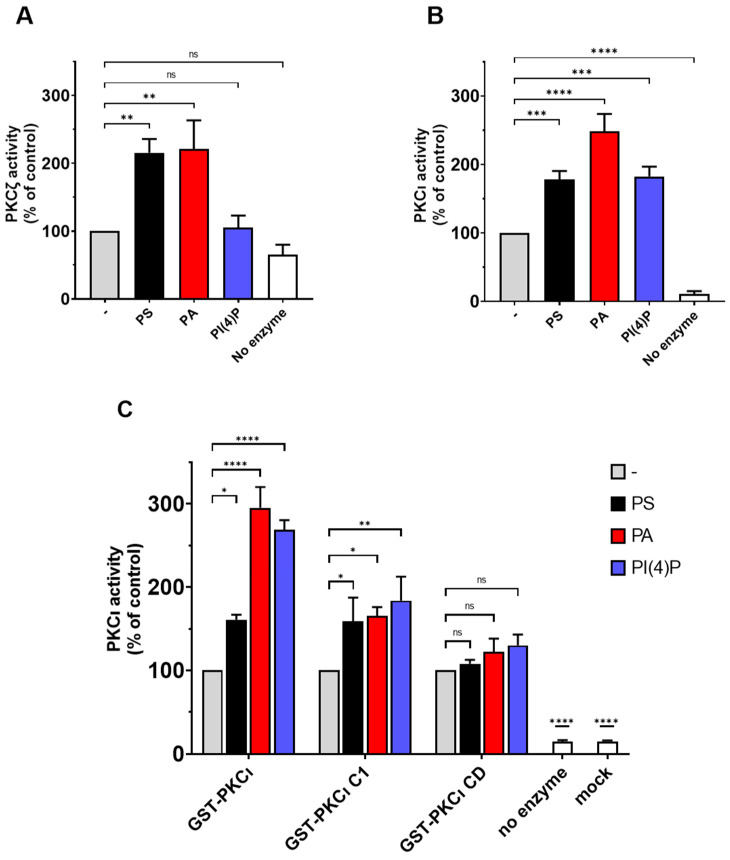
PKCι is activated by PS, PA, and PI(4)P. (**A**) The activity of commercial purified GST-PKCζ was measured in the presence of 50-µg/mL PS or PA or PI(4)P. A complete reaction without an enzyme is considered as the negative control. (**B**) The activity of commercial purified GST-PKCι was measured in presence of 50-µg/mL PS or PA or PI(4)P. A complete reaction without an enzyme is considered as the negative control. (**C**) The activity of in-house purified GST-PKCι and deletion mutants was measured in presence of 0.5-ng/mL PS or PA or PI(4)P. Mock purification and no enzyme conditions are used as negative controls. Data are the mean ± SEM of at least 4 independent experiments performed in triplicate. A single, double, triple and four asterisks denote their significance of *p*-value ≤ 0.05, ≤ 0.01, ≤ 0.001 and ≤ 0.0001 respectively, ns mean No significant.

To evaluate the effect of lipid binding on the catalytic activity of human aPKC, we performed kinase activity assays using highly purified commercial PKCι and PKCζ, incubated with PS, PA, and PI(4)P at a final concentration of 50 µg/mL. Following, the ADP produced was detected using the ADP-Glow luminescence kit. Those assays were run following the preoptimised conditions suggested by the provider; in those conditions, the basal PKCζ activity is quite low. This might be due to the low concentrations of the enzyme (0.1 ng/µL of PKCζ) (four times less enzyme when compared to PKCι—0.4 ng/µL). In similar experimental conditions, the PKCι activity is evidently higher compared to the background (Supplementary Figure S1). However, we can easily observe the expected stimulation of PKCζ in the presence of either PA or PS, while PI4P is ineffective (Figure 4A). Those data are in line with previous reports in the literature [19,27]. 

Conversely, we can easily measure the basal activity of unstimulated PKCι, which is at least 10x when compared to the background. The PKCι basal activity is strongly stimulated by PA, followed by PS (Figure 4B). Interestingly, PI(4)P acts an allosteric activator selective for PKCι, as we observed no activation of highly purified commercial PKCζ by this lipid (Figure 4A,B). These data suggest that the previously reported binding of aPKC to PS, PA, and PIPs enhanced enzyme activity putatively, promoting the switch to the open more active conformation. 

To further investigate the domains involved in lipid-mediated PKCι activation, we performed the kinase activity assays by using in-house purified full-length GST-tagged PKCι and its truncated forms (PKCι C1 and PKCι CD) in the presence of PS, PA, or PI(4)P. Similar to what we observed before, full-length GST-PKCι is strongly activated with PA and PI(4)P, whereas PS activation is still significant but less strong (Figure 4C). The CD is not further activated by lipid mixes in vitro [11]. Even though we noticed some binding of PI(4)P to the PKCι CD mutant, no further activation of the CD was detected in kinase activity assays. The PKCι C1 mutant, which lacks the PB1 and PSR, is considerably inhibited by the C1 domain [12]. The PKCι C1 mutant retained the ability to respond to PA and PI(4P), indicating that the PSR is not required for the activation by these lipids (Figure 4C). Together, the results indicate that the activation by PA and PI(4) is linked to the release of autoinhibition by the C1 domain.

## 3. Discussion

Among the three PKCs subfamilies, the aPKCs (PKCζ and PKCλ/ι) do require neither calcium nor DAG for their activation, but many shreds of evidence indicate that their interactions with lipids may contribute to the control of aPKCs localisation and activity. In order to further understand their mechanisms of regulation and explore their isotype-specific lipid activators, the present work aims to estimate the lipid-binding specificity and activation of the human aPKCs isozymes through a systematic approach.

By performing lipid overlay assays, we observed that aPKCs can selectively bind PA and PS, and this binding results in a relevant increase in aPKCs activity. Those findings are in line with previous results obtained with a lipid motility shift assay [19]. Previously, our group demonstrated that DGKα-produced PA is required to localise the aPKCs to the plasma membrane, where their activity leads to cytoskeletal remodelling and membrane ruffles formation, two essential processes required for cell migration [14,15,21]. These findings indicate that PA provides a key signal to recruit and activate aPKCs at specific membrane compartments. This PA can be derived from DAG through DGK activity or else from PC through phospholipase D (PLD) hydrolysing activity. Indeed, through PA-mediated mechanisms, PLD modulates small GTPases, playing an essential role in membrane homeostasis and cytoskeletal remodelling [33], such as antigen-stimulated membrane ruffling [34]. Interestingly, PLD-generated PA is required for sorbitol-induced activation of aPKCs and GLUT4 translocation/glucose transport [35]. Besides PA, we identified binding to PS, a lipid constitutively present at membranes. We hypothesise that PA, together with PS, recruits and activates aPKC to specific membrane domains. In this manner, DGKα and PLD, modulating PA availability, may be potential regulators of aPKCs localisation and activation. However, the information regarding the spatial and temporal PA distribution in subcellular compartments is still limited. Nishioka et al., using a Phosphatidic Acid indicator (Pii) biosensor based on the FRET technique, observed a divergence in PA content among various cell types and an individual heterogeneity within the same cell line [36]. The authors reported that, upon EGF stimulation, PA level increases rapidly at the plasma membrane, and it seems that this PA production is due mostly to PLD rather than DGK [36]. Similar results were obtained by Zhang and colleagues using a Phosphatidic Acid biosensor with Superior Sensitivity (PASS), and interestingly, their data seem to suggest that EGF triggers a sequential activation of PLD and DGK in distinct membrane nanodomains [37]. 

Interestingly, our findings demonstrated that, unlike PKCζ, PKCι interacts directly with phosphatidylinositol monophosphates (PI(3)P, PI(4)P, and PI(5)P) in a specific and dose-dependent manner. This binding is, at least in part, mediated by the PKCι catalytic domain and results in enzyme activation. Polyphosphoinositides derivatives represent a crucial membrane-localised signal in the control of essential cellular processes by driving the subcellular localisation and activation of specific effector proteins [38]. Indeed, they feature specific subcellular localisation with PI(4)P mainly at the plasma membrane and Golgi apparatus; PI(3)P at the plasma membrane, early endosomal surface and autophagosome; and PI(5)P in very low concentrations at the plasma membrane, the nucleus, Golgi complex, and sarco/endoplasmic reticulum [39,40]. They also feature specific biologic functions: PI(3)P induces autophagy [41], whereas PI(4)P is associated with endosomal trafficking, endoplasmic reticulum (ER) export, autophagy, signalling at the plasma membrane, cytokinesis, and actin dynamics [39,42]. The role of PI(5)P is not completely understood, despite many pieces of evidence suggesting its involvement in the cell cycle, stress response, T-cell activation, and chromatin remodelling [43]. 

The biological significance of this differential lipid regulation between these two highly homologous isoforms relies on their distinct functions. Even if PKCζ and PKCι display 72% amino acid sequence homology, several reports demonstrated functional differences among them. PKCζ is more efficiently involved in the NF-κB activation pathway when compared to PKCι/λ [44,45]. Interestingly, PKCι is frequently overexpressed and mislocalised in human tumours when compared to PKCζ. This involvement results in a consequent loss of cell polarity, which represents the first crucial step towards cell motility and invasiveness [45,46]. In particular, it has been reported that PKCι is often mislocalised to the cytoplasm and the nucleus of the transformed cancer cells [47,48,49,50], but fascinatingly, despite the loss of its restricted localisation within the membrane, PKCι seems to remain in complex with PAR-6 in tumour cells [51,52,53], indicating that this association, along with the PKCι activity, is somehow important for the maintenance of the cancer cell phenotypes [51,54,55,56].

While this work was in preparation, Dong et al. demonstrated that PKCζ is capable of PI(4)P binding only when engaged in a complex with PAR-6, which unmasks the polybasic PSR. This PKCζ-PI(4)P binding is important for the localisation of the complex but not for enzyme activity [27]. Remarkably, our results suggest the existence of a further binding site in the PKCι catalytic domain that is not affected by the phosphorylation position on the inositol ring. While full-length PKCι readily interacted with phosphatidylinositol monophosphates, only the construct comprising the isolated CD of PKCζ showed an interaction with phosphatidylinositol monophosphates. This finding suggests that the CD of both isoforms possess the ability to bind phosphatidylinositol monophosphates but that differences at the N-terminal region hinder the interaction of full-length PKCζ with the phosphatidylinositol monophosphates. In line with this hypothesis, the removal of N-terminal regulatory domains enables PI(4)P and PI(3)P binding to the PKCζ catalytic domain (Figure 3D,E). The small differences in the ability to interact with phosphatidylinositol monophosphates suggest that PKCζ would require binding to PS or PA to “open” the structure of the kinase and expose the catalytic domain that holds the binding site to phosphatidylinositol monophosphates. In a physiological context, those binding sites could be exposed upon PB1 binding to proteins as PAR-6. However, a constitutively active truncated version of PKCζ consisting of the catalytic domain is normally expressed in neuronal cells and is potentially localised by PIPs [57]. 

Though further studies are yet to be conducted regarding this binding, we can speculate that PI(3)P and PI(4)P binding may contribute to the reported recruitment of PKCι at specific membrane compartments, such as the reported localisations at lysosomes [58] or the apical domains of epithelial cells [50]. In the case of PKCζ, it may require recruitment by other lipids, and the binding to phosphatidylinositol monophosphates may support the activity once the protein is recruited to the specific membrane location.

The lipid overlay assay used in our work is a very stringent assay in which the protein must remain bound for the relatively long period of washings; therefore, it detects only high-affinity interactions with relatively low off rates. Indeed, even if PI(3,4,5)P_3_ has been reported as aPKC activator [25,59], we and others were unable to detect any binding suggesting an indirect interaction between aPKC and PI(3,4,5)P_3_ [26]. We also observed no direct binding of aPKC to CE, which was reported to bind PKCζ [22], resulting in recruitment to lipid raft and enzyme activation [60,61]. Recently, by using CE-binding assays and lipid vesicle-binding assays, Wang and colleagues demonstrated that PKCζ can bind to C16:0 CE in a specific manner [18,23]. Similarly, recent studies highlighted a specific interaction between aPKCs and S1P, which is a bioactive lipid obtained by the deacylation of ceramide [24]. In contrast with these data, our approach by lipid overlay assay did not reveal any binding between PKCζ or PKCι and CE or S1P. The discrepancies between our data and those of others could be due to the higher off rates of the interactions with CE or S1P. Alternatively, it is possible that our solid-phase/overlay binding assays may not be suitable to identify proteins that bind to ceramide [62], possibly due to the different conformations of ceramide integrated into a lipid membrane compared to a solid phase. Moreover, to perform the overlay assay, Wang et al. used PKCζ proteolytic fragments, while we used full-length proteins where the CE-binding site may be hidden, as shown for the PI(4)P-binding site. All in vitro studies have limitations, because it is not easy to recapitulate all lipid components and other protein interacting partners in the test tube. Therefore, the study may miss some relevant lipids and protein partners that may be physiologically significant. On the other hand, the assays reported here in two different binding formats and in activity assays were a strong indication that the aPKCs can bind with high affinity and high selectivity to the identified lipids and that they can regulate the activity of PKCι.

Whereas PS is considered to bind to the C1 domain, S1P [24] and PIPs bind to the catalytic domain. The binding of lipids at two different sites on aPKCs provides a means for synergistic binding when two lipids are present. The binding site for PIPs on the catalytic domain has not been determined. However, we can exclude the substrate/pseudosubstrate-binding site as a possible interaction site for PIPs, because the binding there would compete with substrate binding and would be inhibitory. We can speculate that S1P and PIPs could bind at the same region on the small lobe of the catalytic domain where the C1 domain binds. In such a scenario, the activation would be promoted by lipids competing for the two sites of the inhibitory interaction of the C1 domain onto the catalytic domain. 

Finally, in the overlay assay, we report the interaction of PKCι to sulfatide. The interaction was comparably lower, and we did not validate the binding using a second methodology. However, we would like to note that human and yeast PDK1 bind sulfatide as well [63], and PDK1 has also been described to bind to PS [64]. The simultaneous binding of upstream kinase PDK1 and its aPKC substrate to sulfatide and PS could potentially be relevant for the phosphorylation of aPKCs at the activation loop during the maturation stage or as a regulatory event.

In brief, through lipid overlay assays and kinase assays, we observed that both PKCζ and PKCι bind to PA and PS, and the sole PKCι also binds to PI(3)P, PI(4)P, and PI(5)P. Moreover, those interactions result in a selective enhancement of aPKC activity. These data suggest a differential regulation of these two highly homologous isoforms by membrane lipids in line with the reported overlapping but different biological roles.

## 4. Materials and Methods 

### 4.1. Reagents

Anti-FLAG M2 for immunoprecipitation is from Sigma Aldrich, St. Louis, MO, USA (A2220). Horseradish peroxidase-labelled anti-DDK (FLAG) tag is from Origene, Rockville, MD, USA (A190-101P). Secondary antibodies HRP-mouse and HRP-rabbit were from Perkin Elmer.

Unless specified, all chemical reagents, including protease inhibitors mix and protein G agarose are from Sigma Aldrich.

### 4.2. Constructs

The FLAG-PKCζ construct used in Figure 1 was kindly provided by Dr Alex Toker (Boston, MA, USA) [20].

Recombinant human PKCι with C-terminal DDK (FLAG) tag purified ≥ 80% from human HEK293 cells used in Figure 2 is from Origene, Rockville, USA (TP305379).

GST-PKCζ, GST-PKCι, and their truncated forms were previously described in Zang H et al. [12].

### 4.3. Protein Purification

For lipid overlay assays with lipid arrays, recombinant proteins were obtained by transfecting 293T cells (5 × 10 cm dishes) with the corresponding constructs using lipofectamine 3000 (Thermo Fisher Scientific, Waltham, MA, USA) according to the manufacturer’s instruction. After 48 h, cells in each plate were lysed in 0.5 mL of lysis buffer (25-mM HEPES, pH 8, 150-mM NaCl, 1% Nonidet P-40, 5-mM EDTA, 2-mM EGTA, 50-mM NaF, 10% glycerol supplemented with fresh 1-mM Na_3_VO_4_, and protease inhibitors) and clarified after centrifugation for 15 min at 12,000 rpm at 4 °C.

FLAG-tagged recombinant proteins were batch-purified by overnight immunoprecipitation with 50-µg antibody against the protein tag and 100-µL protein-agarose beads. After 4 washes in lysis buffer and 2 in phosphate-buffered saline (PBS—137-mM NaCl, 2.68-mM KCl, 4.3-mM Na_2_HPO_4_, and 1.47-mM KH_2_PO_4_, pH 7.3), the immunoprecipitated protein was eluted twice with 100 µL of 0.1-M glycine (pH 3.5) and immediately neutralised with 10× tris buffer saline (0.5-M tris and 1.2-M NaCl, pH 7.4). 

For purification of GST-tagged recombinant proteins, lysis buffer was supplemented with 2-mM dithiothreitol. GST-tagged proteins were batch-purified upon 4 h of immunoprecipitation with 200-µL glutathione-agarose beads (GE healthcare). After 4 washes in lysis buffer and 2 in phosphate-buffered saline (PBS), the immunoprecipitated protein was eluted with 200-µL elution buffer (100-mM Tris HCl, pH 8.0, 10-mM NaCl, 5% glycerol supplemented with fresh 2-mM DTT, and glutathione 10 mM).

Purified proteins were further subjected to SDS-PAGE for purity evaluation and protein quantification against a BSA calibration curve.

### 4.4. Lipid Overlay Assay

Lipid arrays (Echelon Biosciences, Salt Lake City, UT, USA) used in this work are PIP Strip (P6001), Membrane Lipid Strip (P6002), Membrane Lipid Array (P6003), PIP Array (P6100), and Sphingo Array (S6001). After saturation (3% bovine serum albumin and 0.1% Tween 20 in TBS buffer), membranes were incubated overnight with 5 mL of the protein of interest dissolved in the same buffer. After 4 washes with 0.1% Tween 20 in TBS buffer, the lipid-bound protein was detected upon 1-h incubation with the relevant HRP-labelled anti-tag antibody, followed by a further 4 washes. Detection antibodies were visualised and quantified using Western Lightning Chemiluminescence Reagent Plus (Perkin Elmer, Waltham, MA, USA) and a ChemiDoc imager (Bio-Rad, Hercules, CA, USA). The software automatically checks saturation and auto-scaled images to optimise signal/noise.

### 4.5. Cova PIP ELISA Assay

Proteins of interest were diluted in TBS supplemented with 1% BSA (1 µg/mL in a final volume of 100 µL per well) and added to Cova PIP screening plates (provided by Echelon Bioscience, H-6203; H-6204), followed by overnight incubation with gentle agitation at 4°C. 

After 3 washes using 0.1% Tween 20 in TBS buffer, primary antibody anti-GST (Santa Cruz Biotechnology cat # SC-459, 1:1000 dilution in TBS + 1% BSA) was added and incubated at room temperature for 1 h with gentle agitation. Post-incubation, 3 washes with 0.1% Tween 20 in TBS buffer were performed before adding the secondary antibody diluted 1:5000 in TBS + 1% BSA for 1 h at room temperature on a plate shaker. Later, the plates were washed for 5 additional times before adding 100 µL/well of peroxidase substrate: 3,3′,5,5′-Tetramethylbenzidine liquid substrate (TMB) and stopped the reaction by adding 50 µL/well of 0.5-M H_2_SO_4_ when significant blue colour developed. The absorbance was read with a Tecan Spark instrument plate reader at 450-nm wavelength immediately after adding the stop solution.

### 4.6. aPKC Activity Assay

Protein kinase assays were performed using the PKCι Kinase Enzyme System (Promega; Catalogue #: V3751) and PKCζ Kinase Enzyme System (Promega; Catalogue #: V2781) and ADP-Glo Kinase Assay kit (Promega; Catalogue #: V9101). The reaction was performed in a final volume of 25 μL containing 5 μL of stock solution reaction buffer A supplemented with 2.5-µL DTT (final concentration 50 µM); 5-μL active full-length PKCι (final concentration 4 nM) and 5-μL active full-length PKCζ (final concentration 1 nM); 5 μL of CREBtide substrate stock solution; 2.5 μL of ATP (final concentration 50 µM); 2.5 µL of DMSO (10%); 2.5 μL of lipid activator (10×); or PS, PA, and PI(4)P (dissolved by sonication in MOPS; final concentration 50 µg/mL).

The assay was carried out in 96-well luminescent white plates by incubating the reaction mixture at 30 °C for 20 min. After this incubation period, the ADP-Glo^TM^ Reagent was added to simultaneously terminate the kinase reaction and deplete the remaining ATP. The plate was then incubated for 40 min at room temperature before adding 50 μL of Kinase Detection Reagent to convert ADP to ATP and incubated again for a further 30 min at room temperature. The luminescence of the 96-well reaction plate was finally read using the Tecan Spark 10 M Multimode Plate Reader. 

Controls were set up including all the assay components by replacing the enzyme with equal volume of water (negative control). Lipids were replaced with equal volume of MOPS (for both positive and negative controls).

### 4.7. Data Processing and Statistical Analysis

The binding spots obtained on membrane strips or arrays were acquired with the ChemiDoc imager and quantified by using Image lab 6.0 software (Bio-Rad). Data obtained from PIP arrays and membrane arrays were collected as Excel files and analysed using GraphPad Prism 9.0 software. We analysed our data using one-way ANOVA analysis with Dunnett’s multiple comparisons for ELISA assays (Figure 3B,C), and for kinase activation assays (Figure 4A,B). For Figure 4C of the kinase activation assays, we used two-way ANOVA with Dunnett’s multiple comparisons.

## Figures and Tables

**Table 1:** Protein/lipid interactions detected by the lipid overlay assay.

	PKCZ	PKCι
**PI**	−	−
**PI(3)P**	−	++
**PI(4)P**	−	++
**PI(5)P**	−	++
**PI(3,4)P_2_**	−	−
**PI(3,5)P_2_**	−	−
**PI(4,5)P_2_**	−	−
**PI(3,4,5)P_3_**	−	−
**PA**	++	++
**LPA**	−	−
**PC**	−	−
**LPC**	−	−
**PS**	++	++
**PE**	−	−
**PG**	+	+
**DAG**	−	−
**S**	−	−
**S1P**	−	−
**SPC**	−	−
**PHS**	−	−
**CE**	−	−
**SM**	−	−
**SM4**	−	+
**GM1**	−	−

++ strong signal, + weak signal, and − no signal. PKC: protein kinase C, PIP: phosphatidylinositol monophosphate, PA: phosphatidic acid, LPA: lysophosphatidic acid, PC: phosphatidylcholine, LPC: lysophosphocoline, PS: phosphatidylserine, PE: phosphatidylethanolamine, PG: phosphatidylglycerol, DAG: diacylglycerol, S: sphingosine, S1P: sphingosine-1-phosphate, SPC: sphingosylphosphorycholine, PHS: phytosphingosine, CE: ceramide, SM: sphingomyelin, SM4: sulfatide, and GM1: monosialoganglioside.

## Data Availability

The data presented in this study are available by the authors. For any further request contact the corresponding author.

## Supplementary Materials

The following are available online at https://www.mdpi.com/2227-9059/9/1/45/s1.

## Abbreviations

LPA	lysophosphatidic acid
LPC	lysophosphocoline
PI	phosphatidylinositol
PI(3)P	phosphatidylinositol 3-phosphate
PI(4)P	phosphatidylinositol 4-phosphate
PI(5)P	phosphatidylinositol 5-phosphate
PI(3,4)P_2_	phosphatidylinositol 3,4-bisphosphate
PI(3,5)P_2_	phosphatidylinositol 3,5-bisphosphate
PI(4,5)P_2_	phosphatidylinositol 4,5-bisphosphate
PI(3,4,5)P_3_	phosphatidylinositol 3,4,5-trisphosphate
DAG	diacylglycerol
PA	phosphatidic acid
PS	phosphatidylserine
PE	phosphatidylethanolamine
PC	phosphatidylcholine
PG	phosphatidylglycerol
S	sphingosine
S1P	sphingosine-1-phosphate
SM	sphingomyelin
SPC	sphingosylphosphorycholine
PHS	phytosphingosine
CE	ceramide
SM4	sulfatide
GM1	monosialoganglioside
TAG	triacylglycerol
CL	cardiolipin
CH	Cholesterol
