# Supplementary material for: Identification of Key Phospholipids That Bind and Activate Atypical PKCs
Source: Biomedicines. 2021 Jan 6;9(1):45. doi: 10.3390/biomedicines9010045 (PMC7825596; doi:10.3390/biomedicines9010045)
Supplement: Supplementary file 1 [file biomedicines-09-00045-s001.pdf]

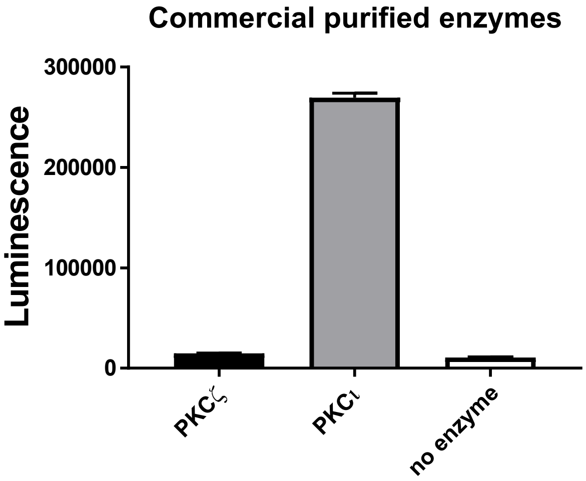

PKCζ	PKCι	no enzyme
14960	273854	11848
14854	273922	10029
15041	261339	10455

Supplementary Figure 01: *PKCζ basal activity is quite low compared to PKCι*

The basal activity of commercial purified GST-PKCζ (0.1 ng/μl) and GST-PKCι (0.4 ng/μl) was measured in the preoptimized conditions of PKCζ enzyme system, using ADP glow kinase assay system and luminescence as a readout of enzyme activity.
